# The association between trimethylamine N-oxide levels and ischemic stroke occurrence: a meta-analysis and Mendelian randomization study

**DOI:** 10.1186/s12883-023-03458-2

**Published:** 2023-11-21

**Authors:** Xinhua Hu, Haiyan Ren, Yuan Cao

**Affiliations:** 1 Department of Neurology, People’s Hospital of Xinjin District, Chengdu, China; 2 Department of Neurology, Shanghai Sixth People’s Hospital Xuhui Branch Affiliated With Shanghai Jiao Tong University, Shanghai, China

**Keywords:** Trimethylamine-N-oxide, Ischemic stroke, Meta-analysis, Mendelian randomization

## Abstract

**Background:**

Trimethylamine-N-oxide (TMAO), an intestinal microbiota-derived choline metabolite, has been found to be associated with ischemic stroke (IS) in more and more studies. However, the causal role of TMAO on IS occurrence remains perplexing.

**Methods:**

We comprehensively screened the related clinical studies on PubMed, Web of Science, and Embase. Case-control and cohort studies that reported the TMAO levels of both IS patients and healthy controls were included, and the risk of bias was assessed according to the criteria by the Centre for Evidence-Based Medicine in Oxford, UK. A meta-analysis of the retrieved publications was performed with a random-effect model to analyze the connection between TMAO levels and IS events. Besides, a Mendelian randomization (MR) analysis was performed to study the causal effect of TMAO on IS, with pooled data of TMAO and IS obtained from genome-wide association studies (GWAS). The following methods were used: MR-Egger, weighted median, inverse-variance weighted, simple mode, and weighted mode. The study has been registered in INPLASY (Registration number: INPLASY2023100027).

**Results:**

Eight cohort or case-control studies covering 2444 cases and 1707 controls were identified. The pooled data indicated that the IS patients tended to have higher TMAO levels compared with the controls (mean difference: 1.97 μM; 95% confidence interval [CI]: 0.87, 3.07; *P* = 0.0005), while distinctive heterogeneity (*I*^*2*^ = 96%, *P* < 0.00001) was observed. Sub-group analysis revealed that the heterogeneity of the studies might be derived from the studies themselves. However, no causal effect of TMAO on IS was observed (*P* > 0.05) in the Mendelian randomization analysis of this study.

**Conclusion:**

We confirmed that IS patients tend to have higher TMAO levels than healthy individuals, while our findings of MR analysis did not support the causal role of TMAO in IS occurrence. Therefore, more studies are required for a better understanding of the relationship between TMAO levels and IS onset.

**Supplementary Information:**

The online version contains supplementary material available at 10.1186/s12883-023-03458-2.

## Background

Stroke is the second leading cause of death nowadays whilst ischemic stroke accounts for 85% of stroke incidences globally [1]. Ischemic stroke (IS) is caused by an abrupt blockage of blood flow that results in damage to the central nervous system and thus disability or death of the patients. IS is always an emergent threat to humans. Increasing evidence indicated that the gut microbiota is closely related to different diseases in the human body, including cardiovascular and cerebrovascular diseases [2]. The gut microbiota can modulate the neuron networks, thereby regulating the progression of stroke, through a complex bidirectional brain-gut axis, in which various metabolites play a key role [3–6].

Trimethylamine-N-oxide is an organic small molecular metabolite, and is converted by gut microbiota from trimethylamine (TMA)——the waste product of TMA-containing dietary nutrients, such as phosphatidylcholine, choline, and carnitine [7]. TMAO can promote platelet hyperreactivity and increase thrombosis risk, and a growing body of research has suggested a close relationship between TMAO and IS [3, 8]. Most researchers reported that TMAO levels are positively correlated to the incidences of IS, and TMAO is potent to be the biomarker of IS [9–14]. However, the results of several studies are controversial, as decreased levels of plasma TMAO in IS patients were also reported [15, 16]. Besides, several reports indicated that increased plasma TMAO concentrations might contribute to poor prognosis of IS patients [17, 18]. Thus, a definite correlation and causal relationship between TMAO levels and IS incidence remains perplexing and requires further research.

In this study, we conducted a meta-analysis based on case-control and cohort studies to investigate the association between TMAO levels and IS occurrence by pooling the current studies, and evaluate the potential of TMAO to be a biomarker that predicts IS incidences. At the same time, the causal effect of TMAO on IS was studied using a two-sample MR method.

## Methods

### Meta-analysis

#### Literature search

Systematic research was performed in PubMed, Web of Science, and Embase for the related studies up until September 2023. Our medical subject heading terms were “(Trimethylamine N-oxide [Title/Abstract] OR TMAO [Title/Abstract]) AND (ischemic stroke [Title/Abstract])” for PubMed, “(TS = (Trimethylamine N-oxide OR TMAO)) AND TS = (Ischemic stroke)” for Web of Science, “((Trimethylamine N-oxide or TMAO) and ischemic stroke).mp. [mp = title, abstract, heading word, drug trade name, original title, device manufacturer, drug manufacturer, device trade name, keyword heading word, floating subheading word, candidate term word]” for Embase. The screened references for the articles were manually checked (Table S1). The publications both in English and Chinese were included, and EndNote 21 was used to remove duplicate articles.

#### Study selection

Studies were included if they were case-control, or cohort studies that reported the TMAO levels of both IS patients and healthy controls. The systematic reviews and the abstracts were excluded. Two reviewers (YC and XH) independently assessed the eligibility of the identified articles, and a third reviewer (HYR) was consulted when disagreements occurred.

The inclusion criteria for the patients in this meta-analysis were: (1) studies including both consecutive patients with first-ever ischemic stroke and controls; (2) symptoms onset less than 24 h; (3) blood sample collected within 24 h post IS onset.

The exclusion criteria for this study were: (1) subjects with myocardial infarction, heart failure, malignant tumor, or other systemic diseases; (2) subjects who had used antibiotics/prebiotics or experienced gastrointestinal symptoms in the past 3 months; (3) systematic reviews, meta-analysis articles, and conference abstracts.

#### Data extraction and processing

Two of the researchers (YC and XH) independently extracted the data including author names, year of the study, country/area, and race of the population, sample sizes of cases and controls, and characteristics of IS cases and controls. A third reviewer (HYR) was consulted when disagreements occurred. The data for TMAO levels expressed as mean ± SD were directly obtained; the data shown as median with interquartile range were converted to mean ± SD using the previously reported method [19]; and the data illustrated as figures were extracted using the software Engauge Digitizer 4.1 (https://markummitchell.github.io/engauge-digitizer/).

#### Assessment of data quality and study risk of bias assessment

The evidence levels of identified studies were assessed according to the criteria by the Centre for Evidence-Based Medicine in Oxford, UK [20]. The quality of each publication was evaluated by bias analysis using Review Manager 5.3. A funnel plot of all included studies, as well as Begg’s test and Egger’s test, was exploited for the assessment of publication bias.

#### Statistical analysis

All the statistical analysis was performed using Review Manager 5.3 and Stata/SE 12.0. Since the obtained data for TMAO levels were continuous, the weighted mean differences with 95% CI were used to compare the difference in TMAO between IS patients and the controls. Statistical heterogeneity among the studies was quantified using the *I*^*2*^ statistic. A random-effect model was used if the studies were heterogeneous, while otherwise, a fixed-effect model was adopted. Subgroup analyses were performed to evaluate the effect of race or sample variations. We performed the sensitivity analyses by excluding one study at a time to estimate the influence of every single study on the overall estimate.

The TMAO levels were extracted as means (SD), and the results of the meta-analysis were presented as weighted mean differences [95% CIs]. Results were considered to be statistically significant when a value of *P* < 0.05 was obtained.

### Mendelian randomization analysis

#### Data source

Summary data for TMAO were obtained from a GWAS of 217 plasma metabolites in the Framingham Heart Study including 2,076 European participants [21]. The single nucleotide polymorphisms (SNPs) of TMAO with suggestive genome-wide significance thresholds (*P* < 5 × 10^–5^) were selected and clumped as instrumental variables (IVs).

Summary data for IS were extracted as the outcome from the IEU GWAS database (https://gwas.mrcieu.ac.uk/) with the trait of “Ischemic stroke”, including 34,217 Europeans (id: ebi-a-GCST006908) [22].

#### Two-sample mendelian randomization

Inverse-variance weighted (IVW) method, the most efficient estimate in the absence of pleiotropy [23], was mainly utilized to evaluate the relationship between TMAO and IS. The random-effects model was used if heterogeneity existed; otherwise, the fixed-effects model was used. MR-Egger, Maximum likelihood, Weighted median, and Weighted mode were also exerted for further validation of the IVW results. The relationship between TMAO SNPs and IS outcome was visualized through a scatter plot. The Wald ratio for single SNPs and their combined effects were illustrated with a forest plot. The horizontal pleiotropy was evaluated by MR-Egger regression and a funnel plot. The heterogeneity was estimated using IVW and MR-Egger analysis. Leave-one-out analysis was conducted to determine the effect of each single SNP on the MR results.

The TwoSampleMR package of R (https://github.com/MRCIEU/TwoSampleMR) was used to perform the statistical analysis, and the results were shown as beta ± SE. A P value less than 0.05 was considered a significant difference.

### Study registration

The protocol of this study has been registered in INPLASY (Registration number: INPLASY2023100027).

## Results

### Meta-analysis

#### Literature search

**Fig. 1:**
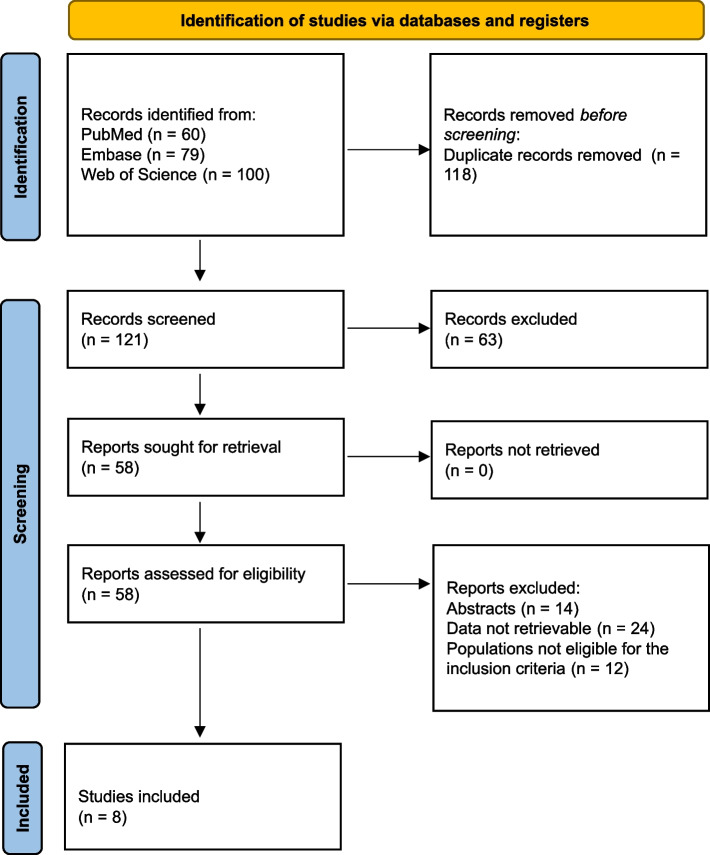
Flow chart illustrating the process of identification, exclusion, and inclusion of the studies for the meta-analysis

A total of 239 records were identified from previous studies, of which 118 were duplicates to be removed. Subsequently, 63 irrelevant studies were excluded by the manual screening of the title and the abstract. Besides the mentioned databases (PubMed, Web of Science, and Embase), we have also performed a literature search in Google Scholar and got about 6,150 results, which were duplicate or irrelevant and not included here. After the full-text assessment, we finally obtained 8 publications including 4,151 cases (2,444 for IS patients, and 1,707 for the controls) eligible for the meta-analysis (Fig. 1).

Several studies appeared to meet the inclusion criteria but were excluded due to various reasons. Zhu et al. [24] reported the association of plasma TMAO levels with post-stroke cognitive impairment, and the TMAO concentrations of 256 IS patients were detected, but unfortunately, no healthy control was included. Similar situations also occurred in the other two studies [25–27]. In a study by Yin et al. [15], the TMAO concentrations were compared between the IS patients and the asymptomatic control, but all the included subjects had atherosclerosis which might affect the overall results. Besides, in the study by Liu et al. [28], 412 identified stroke cases and 412 controls were studied, while both IS and non-ischemic stroke cases were combined, and the data for IS only were not available. In a retrospective study about two prospective cohorts [29], CHS conducted in 1989 to 1990 and MESA conducted in 2000-2002, the researchers investigated the association between plasma TMAO and incident IS, but the data of TMAO concentrations for the controls or the IS cases were not available.

#### Data extraction and quality assessment

The characteristics of identified studies are summarized in Table 1. Most of the studies [9–14, 16] were assessed as having a low risk of bias. The acquisition of the diagnostic information was mostly from consecutive patients, and can be regarded as randomization and blinding of the participants. In the study by Haak et al. [16], not all the data were presented; therefore, this study was of high risk in data attrition and reporting, as illustrated by Figure S1.

**Table 1:** Characteristics of the included studies of the meta-analysis

Study	Year	Study design	Population (IS patients)	Comparison (Healthy controls)	Intervention (Measurements)	Matching^a^	Quality score^b^
Haak et al	2021	a sub-study of a randomized trial	349 ischemic and hemorrhagic stroke patients	51 healthy non-hospitalized age- and sex-matched controls	Plasma TMAO	1, 2, 4, 5, 6, 7, 8	★★★★☆
Liu et al	2017	a cohort study	80 atherosclerotic ischemic stroke patients	40 healthy subjects	Serum TMAO	1, 2	★★★★☆☆☆
Rexidamu et al	2019	a cohort study	255 first-ever ischemic stroke patients	255 age and gender-matched healthy volunteers	Serum TMAO	1, 2, 3, 4, 5, 6, 8	★★★★★★☆
Schneider et al	2020	a case–control study	193 patients suffering from ischemic stroke (onset < 24 h)	100 control patients with less than two cardiovascular risk factors	Plasma TMAO	1, 2, 4, 5, 6, 7, 8	★★★★★☆☆
Sun et al	2021	a case–control study	953 ischemic stroke cases	953 control subjects	Plasma TMAO	1, 2, 3, 4, 5	★★★★★★☆
Tan et al	2020	a cohort study	204 acute ischemic stroke patients	108 healthy controls	Plasma TMAO	1, 2, 4, 5, 6, 7, 8	★★☆☆☆☆☆
Wu et al	2020	a cohort study	377 acute ischemic stroke patients	50 healthy controls	Plasma TMAO	1, 2, 3, 4, 5, 6, 8	★★★★☆☆☆
Zhang et al	2021	a cohort study	351 patients with first-ever ischemic stroke	150 age and gender-matched healthy volunteers	Plasma TMAO	1, 2, 3, 4, 5, 6, 7, 8	★★★★★★☆

^a^Matching: 1 = age; 2 = gender; 3 = BMI; 4 = prior vascular risk factors (hypertension, diabetes, hypercholesterolemia, atrial fibrillation, et al.); 5 = life style (smoking, alcoholism, diet); 6 = pre-stroke treatment (antihypertensive, anticoagulant, statins, et al.); 7 = acute treatment (IV thrombolysis, mechanical thrombectomy); 8 = stroke characteristics (NIHSS, stroke volume)

^b^The quality score was obtained by bias assessment, in which low bias risk was presented as “★”, while unclear risk of bias was illustrated as “☆”, and no pentagram was obtained for the study domains with high risk of bias. The domains for bias analysis were shown in Figure S1

#### Meta-analysis of TMAO levels in IS patients and the controls

**Fig. 2:**
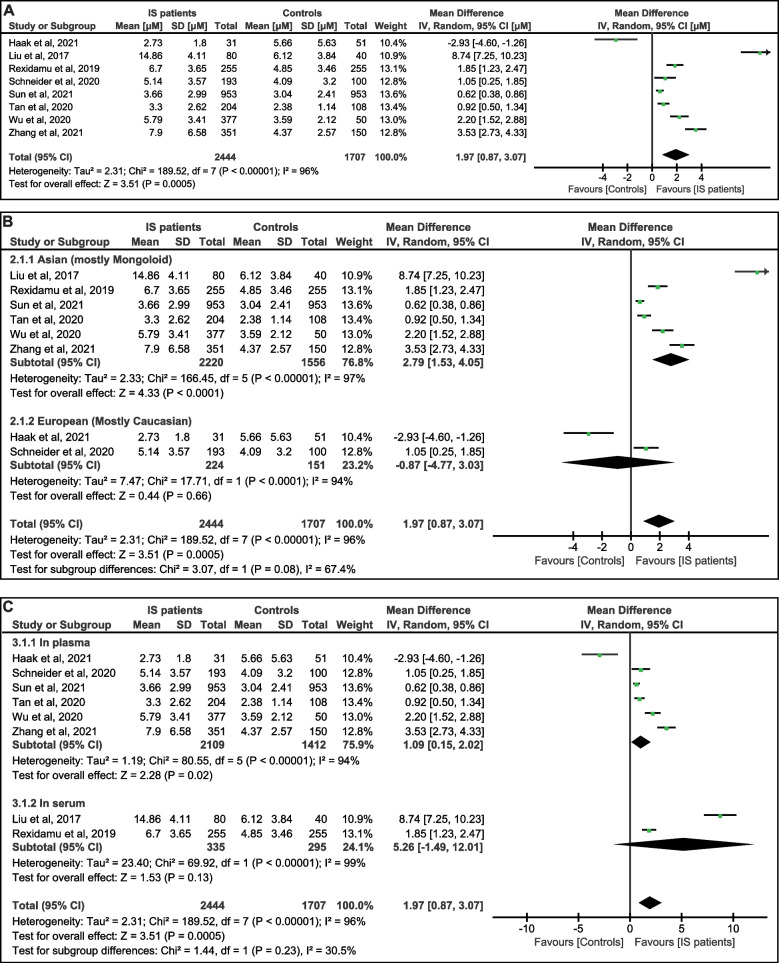
Meta-analysis of the association between TMAO levels and the IS patients. **A** The forest plot and meta-analysis of the pooled data in all included studies. The extracted data were means (SD) in μM, and the analysis results were weighted mean difference [95% CIs]. **B** Forest plot and sub-group meta-analysis that compares the races. **C** Forest plot and sub-group meta-analysis that compares the sample types

According to the data pooled from eight studies including 4,151 cases, the IS patients tended to have higher levels of TMAO in their blood than the controls (mean difference: 1.97 μM; 95% CI: 0.87, 3.07; *P* = 0.0005) (Fig. 2A). To be noted, significant heterogeneity among these studies (*I*^*2*^ = 96%, *P* < 0.00001) was observed, thus we exploited the random-effect model for the data analysis.

In addition, we performed two sub-group assessments by comparing the data from different areas or samples. In the six studies in Asia, where most individuals were Mongoloid, the TMAO levels of IS patients were higher than that of the controls (mean difference: 2.79 μM; 95% CI: 1.53, 4.05; *P* < 0.0001); while in the two studies in Europe, where most cases were Caucasian, no significant difference was observed (mean difference: -0.87 μM; 95% CI: -4.77, 3.03; *P* = 0.66) (Fig. 2B). This phenomenon suggested the possible influence of the areas or the races. However, only two study was included in the sub-group of Europe (mostly Caucasian), which was not enough for a conclusive analysis.

Of the selected studies, six detected the TMAO concentrations in the plasma, while two quantified TMAO in the serum. As for the plasma sub-group, the IS patients possessed statistically higher TMAO levels (mean difference: 1.09 μM; 95% CI: 0.15, 2.02; *P* = 0.02). No significant difference was exhibited in the serum sub-group (mean difference: 5.26 μM; 95% CI: -1.49, 12.01; *P* = 0.13) (Fig. 2C).

Of note, one study [16] showed lower TMAO levels in IS patients, and one study [14] reported markedly higher TMAO concentrations than the rest of the research. Heterogeneity existed in both overall and sub-group analysis. Even though the results of the two sub-groups analysis vary a lot, the tests for sub-group differences indicated no significant variations (Asian sub-group vs European sub-group, *P* = 0.08; plasma sub-group vs serum sub-group, *P* = 0.23) (Fig. 2B, C), which suggested that the heterogeneity might come from the variations between each study.

#### Sensitivity analysis

**Fig. 3:**
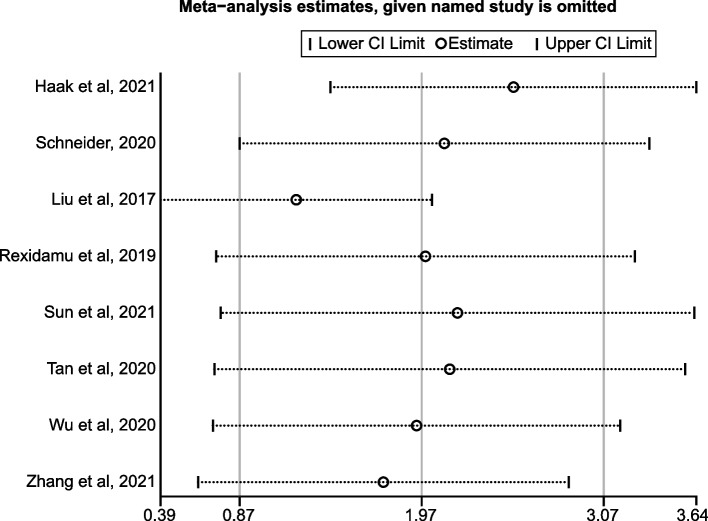
The sensitivity analysis for meta-analysis of all quantitative studies using the leave-one-out method

The influence of each study on the overall results was investigated by excluding every single study at a time in data analysis. As shown in Fig. 3, we found that removing any of the studies did not change the analysis result or the trend of mean difference of TMAO between the IS patients and the controls. Even though the study by Liu et al. [14] reported notably higher TMAO levels compared with the others, excluding this study did not change the significance of the overall analysis. Besides, although Haak et al. got an unexpected finding that was controversial to the other trials, their results exhibited little impact on the final results of this meta-analysis. These findings indicated that the pooled results were consistent and could barely be altered by a single study, despite that great heterogeneity existed in present studies.

#### Publication bias

**Fig. 4:**
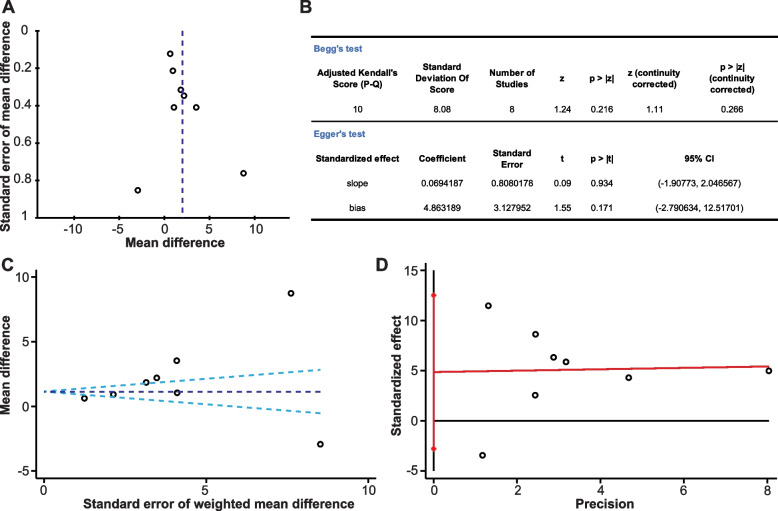
Evaluation of publication bias of included studies for meta-analysis. **A** Funnel plots of all included studies. **B** The results of Begg’s test and Egger’s test. **C** The plot of Begg’s test. **D** The plot of Egger’s test

A funnel plot of the studies included in this meta-analysis seemed symmetrical by visual observation, indicating no perceptible bias (Fig. 4A). We used the random-effect model that assumes these studies had different true effects due to between-study heterogeneity, and thus no pseudo 95% CI line was added. Begg’s test and Egger’s test were also conducted and suggested no publication bias (Begg’s test, *P* = 0.266; Egger’s test, P_slope_ = 0.934, P_bias_ = 0.171) (Fig. 4B-D).

### Mendelian randomization analysis

#### Selection of SNPs

The SNPs of TMAO and IS included in this study are shown in Table S2. We extracted TMAO SNPs from the GWAS data of the Framingham Heart Study of Europeans [21], and after clumping (*P* < 0.001), 53 TMAO SNPs were finally retrieved. Subsequently, 52 SNPs for IS were extracted from a European-only and transancestral GWAS [22].

#### MR statistical results

**Fig. 5:**
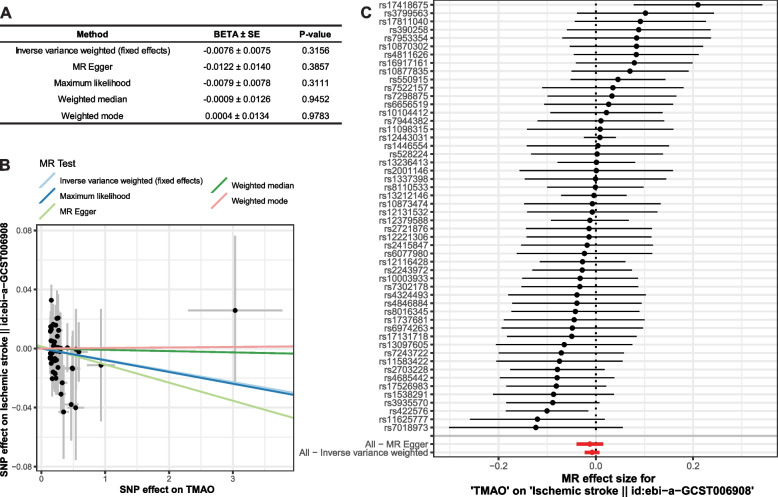
MR analysis of the causal effect of TMAO on IS. **A** MR results calculated using five methods. **B** The scatter plot visualizing the relationship between TMAO SNPs and IS SNPs. **C** The forest plot displaying the Wald ratio for single SNPs and their combined effects

We found that TMAO did not show a statistically significant causal effect on IS, according to the MR analysis through the IVW method (beta: -0.0076, SE: 0.0075, *p* = 0.315) (Fig. 5A). As no heterogeneity was observed, a fixed-effects model for IVW was used. This was also confirmed by other methods such as MR-Egger method (beta: -0.0122, SE: 0.0140, *p* = 0.386), Maximum likelihood method (beta: -0.0079, SE: 0.0078, *p* = 0.311), Weighted median method (beta: -0.0009, SE: 0.0126, *p* = 0.945), and Weighted mode method (beta: -0.0004, SE: 0.0134, *p* = 0.978).

The relationship of TMAO SNPs against that of IS was depicted using a scatter plot **(**Fig. 5B), and the slopes of the lines correspond to the estimated causal effect for each method. Even though an inverse causal effect seemed to exist in the methods of Inverse variance weighted, Maximum likelihood, and MR Egger, the effect was not statistically significant (Fig. 5A, B). We displayed the Wald ratio for single SNPs and their combined effects using the IVW method through a forest plot (Fig. 5C, Table S3).

#### Sensitivity analysis

Heterogeneity was not observed through the IVW (*p* = 0.396) or MR-Egger (*p* = 0.429) method (Table S4). The intercept from the MR-Egger regression analysis was not significant (*p* = 0.0691) (Table S5), which indicated no significant pleiotropy. In the leave-one-out analysis, no single SNP was driving the association (Figure S2). The funnel plot exhibited a symmetrical distribution of SNP loci, indicating the reliability of this MR analysis (Figure S3). The radial MR analysis suggested no significant outlier SNP (Figure S4).

## Discussion

In the meta-analysis of eight studies involving 4,151 cases, we confirmed the positive correlation between TMAO levels and IS onset. The IS patients tend to have higher TMAO levels than the healthy people in most cases. However, the Mendelian randomization analysis in this research indicated no causal effect of TMAO on IS.

Noticeable between-study heterogeneity existed in the meta-analysis, probably due to the differences in age, race, geographic location, or dietary habits of the individuals in the cohorts. Although most research indicated higher TMAO levels in IS patients, some reported controversial results: In the work by Haak et al. [16], the TMAO levels of IS patients were lower than that of the control group. Haak et al. speculated that this might be attributed by the following factors: (1) TMAO levels could be altered by treatment with intravenous thrombolytic therapy; (2) the amphipathic TMAO molecules might have entered cells, reducing the detection value in the system circulation, according to a previous report [8]; (3) TMAO levels were reported to decrease in the hours and days following stroke [11, 30]. Similar results have also been reported by Yin et al. [15], and they found that the patients of the acute stroke group exhibited lower TMAO levels than the individuals of the asymptomatic control group. The authors explained that they selected patients with asymptomatic atherosclerosis, and the TMAO level might have decreased with the onset or treatment of IS, which requires further clarification.

The two opposite phenomena observed in the above studies are likely influenced by a variety of factors including, but not limited to, the timing of testing, the underlying medical condition, medications being used, and dietary differences. The TMAO concentrations actually vary dynamically over time and can be affected by the time point for detection or the iatrogenic factors. It has been shown that the blood TMAO levels may be at a high level within 24 h of IS onset, and then gradually decrease [11, 30]. Treatment with antibiotics or intravenous thrombolytic therapy could also change the production of TMAO [3, 11, 16]. Since TMAO is a metabolite produced by the gut microbiota, its production can be greatly affected by the host diet. This may partially explain the heterogeneity existing in different studies of the meta-analysis. A high-fat diet or consumption of eggs and choline supplements will increase the concentration of TMAO in the blood [3, 31–33]. Therefore, if TMAO is to be used as a predictor of IS, not only the timing of testing needs to be consistent, but also the medical/medication history as well as the lifestyle and dietary habits of the tested individuals need to be taken into account.

It has been reported that TMAO could act as an “accomplice” in atherosclerosis, thrombosis, arterial injury, and neurological damage by promoting macrophage foam cell formation, modulating platelet hyperactivity, disrupting cholesterol metabolism, enhancing inflammation, et al. [3, 34]. TMAO can thus be a molecule that bridges diet, gut microbiota, and IS occurrence. However, the definite role of TMAO in the development and pathogenesis of stroke remains unclear. Our MR analysis in this study did not confirm the causal effect of TMAO on IS, either. This result was similar to a previous report about gut microbiota–dependent metabolites and cardiometabolic disease [35]. Even though a lot of evidence confirmed the association of TMAO and IS, our MR results did not support the causal relationship. All selected SNPs were clumped using the European population from the 1000 Genomes Project [36] (*P* < 0.001), however, we cannot guarantee that our results were not affected by unmeasured confounders. There is the possibility that TMAO and IS share a similar genetic basis leading to strong clinical associations between them.

The pathogenesis of IS is quite complex and closely related to multiple risk factors, including personal characteristics like age, gender, race/ethnicity, and heredity; the underlying disease like hypertension, cardiac disease, particularly atrial fibrillation, diabetes mellitus, hypercholesterolemia; and the lifestyles such as cigarette smoking and alcohol abuse [37]. IS occurrence can be influenced by the gut microbiota through the bidirectional brain-gut axis: the gut microbiota can increase the risk of a cerebrovascular event by its metabolites, while conversely, stroke can interrupt the integrity of epithelial barrier and result in dysbiosis of gut microbiota [4]. TMAO, as a by-product of the intestinal microflora, can enhance the aggregation of platelets and contribute to the hypercoagulable states and has been reported to be directly associated with poor IS outcomes [3, 8, 38, 39]. Be noted, there are similar studies that evaluate the relationship between the reoccurrence of stroke and baseline TMAO levels and discovered that elevated TMAO levels are related to recurrent stroke independent of dual-antiplatelet therapy, intensive lipid-lowering therapy at discharge, and low inflammation on admission [40], particularly in patients with small artery occlusion subtype [41].

In this study, we focused on the relationship between TMAO and IS. In fact, a large number of other small molecule metabolites may be associated with the course of IS. For example, Chen et al. compared control and mild IS patients over 3 years and found significant differences (*P* < 0.001) in the blood biochemistry biomarker high-density lipoprotein, and the small molecule metabolites lactic acid, pyruvic acid, and TMAO [42]. Liu et al. compared the levels of TMAO and its precursors carnitine, choline, betaine, and trimethyl lysine (TML) in control subjects and patients with various types of stroke, and found significant differences only for TMAO [28]. Kijpaisalratana et.al used a stroke case-cohort nested within the Reasons for Geographic and Racial Differences in Stroke (REGARDS) study and identified that the plasma metabolite gluconic acid was associated with IS among Black participants but not White participants [43]. Taken together, despite the large number of other measures associated with IS, TMAO has been reported most frequently and with more consistent conclusions. However, whether TMAO is the most significant compared to other metabolites requires further exploration.

There are several limitations to this research. First, the lack of clinical studies limited the generalisability of our results to different ethnicities and different sample types, as only two studies of Europeans were included in the meta-analysis, and two studies used serum samples. Second, to maximize the sample size we combined studies with different study designs, and there are differences in the cohort groups, inclusion criteria, and matching strategies among these included studies, which might cause the significant between-study heterogeneity in the meta-analysis. In addition, even though we did not observe the causal association between genetically increased TMAO and IS, we cannot conclude whether the causal association exists or not, or whether TMAO and IS share a genetic basis.

## Conclusions

In summary, the meta-analysis suggests that higher TMAO levels are associated with IS occurrence, while the MR results do not support a causal effect of TMAO with IS. More studies are required to explain further insight into the relationship between TMAO levels and IS occurrence.

### Supplementary Information

**Additional file 1: Table S1.** Search stratages for literatures applied in the meta-analysis. **Table S2.** Genetic IVs for TMAO. **Table S3.** Estimation of Wald ratio between each SNP related to TMAO and risk of IS. **Table S4.** Heterogeneity analysis using Cochran’s Q test. **Table S5.** Horizontal pleiotropy analysis using the intercept of MR-Egger regression test. 

**Additional file 2:** **Fig. S1.** Quality assessment of the included studies for meta-analysis. **Fig. S2.** Leave-one-out analysis indicated that no single SNP was driving the association of MR analysis. **Fig. S3. **The funnel plot for visually inspection for horizontal pleiotropy of MR analysis. **Fig. S4.** The Radial plots for classification of outliers in the SNPs.

## Data Availability

All data generated or analysed during this study are included in this published article and its supplementary information files.

## Abbreviations

TMAO: Trimethylamine-N-oxide
IS: Ischemic stroke
MR: Mendelian randomization
GWAS: Genome-wide association studies
CI: Confidence interval
TMA: Trimethylamine
SNPs: Single nucleotide polymorphisms
IVW: Inverse-variance weighted
TML: Trimethyl lysine
REGARDS: The Reasons for Geographic and Racial Differences in Stroke
