# Supplementary material for: The association between trimethylamine N-oxide levels and ischemic stroke occurrence: a meta-analysis and Mendelian randomization study
Source: BMC Neurol. 2023 Nov 21;23:413. doi: 10.1186/s12883-023-03458-2 (PMC10662484; doi:10.1186/s12883-023-03458-2)
Supplement: Supplementary file 2 — Additional file 2: Fig. S1. Quality assessment of the included studies for meta-analysis. Fig. S2. Leave-one-out analysis indicated that no single SNP was driving the association of MR analysis. Fig. S3. The funnel plot for visually inspection for horizontal pleiotropy of MR analysis. Fig. S4. The Radial plots for classification of outliers in the SNPs. [file 12883_2023_3458_MOESM2_ESM.docx]

**Supplementary information**

**Contents**

1. Description of the statistical model of the Mendelian randomization analysis.
2. Fig. S1 Quality assessment of the included studies for meta-analysis.
3. Fig. S2 Leave-one-out analysis indicated that no single SNP was driving the association of MR analysis.
4. Fig. S3 The funnel plot for visually inspection for horizontal pleiotropy of MR analysis.
5. Fig. S4 The Radial plots for classification of outliers in the SNPs.

**Description of the statistical model of the Mendelian randomization analysis.**

Mendelian randomization (MR) is a method to address the causal effects of exposures to outcomes. MR follows the principles based on Mendel’s laws of inheritance and instrumental variable estimation methods and makes it possible to infer causal influences while unobserved confounding factors exist. Genetic variation, usually implemented using single nucleotide polymorphisms (SNPs), is applied in MR based on instrumental variable (IV) analysis. [1-3]

In this study, we tried to evaluate the causal effect of TMAO on IS. Therefore, TMAO was regarded as the exposure, while IS was regarded as the outcome. In order to eliminate the interference of confounding factors, we extracted SNPs of TMAO from the GWAS database [4] with P-values less than 5 × 10^-5^, and performed linkage disequilibrium (LD) clumping; then the extracted SNPs were used as instrumental variables (IVs). The SNPs of IS were then extracted from the IEU GWAS database (id: ebi-a-GCST006908) [5]). Next, the SNPs of both the exposure (TMAO) and outcome (IS) were harmonized for MR analysis. Five methods (IVW, MR-Egger, Maximum likelihood, Weighted median, and Weighted mode) were employed in the MR analysis of this study with the aim of minimizing potential bias. The relationship between TMAO SNPs and IS outcome was visualized through a scatter plot. The Wald ratio for single SNPs and their combined effects were illustrated with a forest plot. The horizontal pleiotropy was evaluated by MR-Egger regression and a funnel plot. The heterogeneity was estimated using IVW and MR-Egger analysis. Leave-one-out analysis was conducted to determine the effect of each single SNP on the MR results.

The Mendelian randomization analysis in this study was performed using the TwoSampleMR package of R (<https://github.com/MRCIEU/TwoSampleMR>).

**Reference**

1. Burgess S, Thompson SG: **Mendelian randomization: methods for using genetic variants in causal estimation**: CRC Press; 2015.

2. Burgess S, Thompson SG: **Mendelian randomization: methods for causal inference using genetic variants**: CRC Press; 2021.

3. Sanderson E, Glymour MM, Holmes MV, Kang H, Morrison J, Munafò MR, Palmer T, Schooling CM, Wallace C, Zhao Q *et al*: **Mendelian randomization**. *Nature Reviews Methods Primers* 2022, **2**(1):6.

4. Rhee EP, Ho JE, Chen MH, Shen D, Cheng S, Larson MG, Ghorbani A, Shi X, Helenius IT, O'Donnell CJ *et al*: **A genome-wide association study of the human metabolome in a community-based cohort**. *Cell Metab* 2013, **18**(1):130-143.

5. Malik R, Rannikmae K, Traylor M, Georgakis MK, Sargurupremraj M, Markus HS, Hopewell JC, Debette S, Sudlow CLM, Dichgans M *et al*: **Genome-wide meta-analysis identifies 3 novel loci associated with stroke**. *Ann Neurol* 2018, **84**(6):934-939.

6. **Oxford Centre for Evidence-Based Medicine: Levels of Evidence (March 2009)** [<https://www.cebm.ox.ac.uk/resources/levels-of-evidence/oxford-centre-for-evidence-based-medicine-levels-of-evidence-march-2009>]


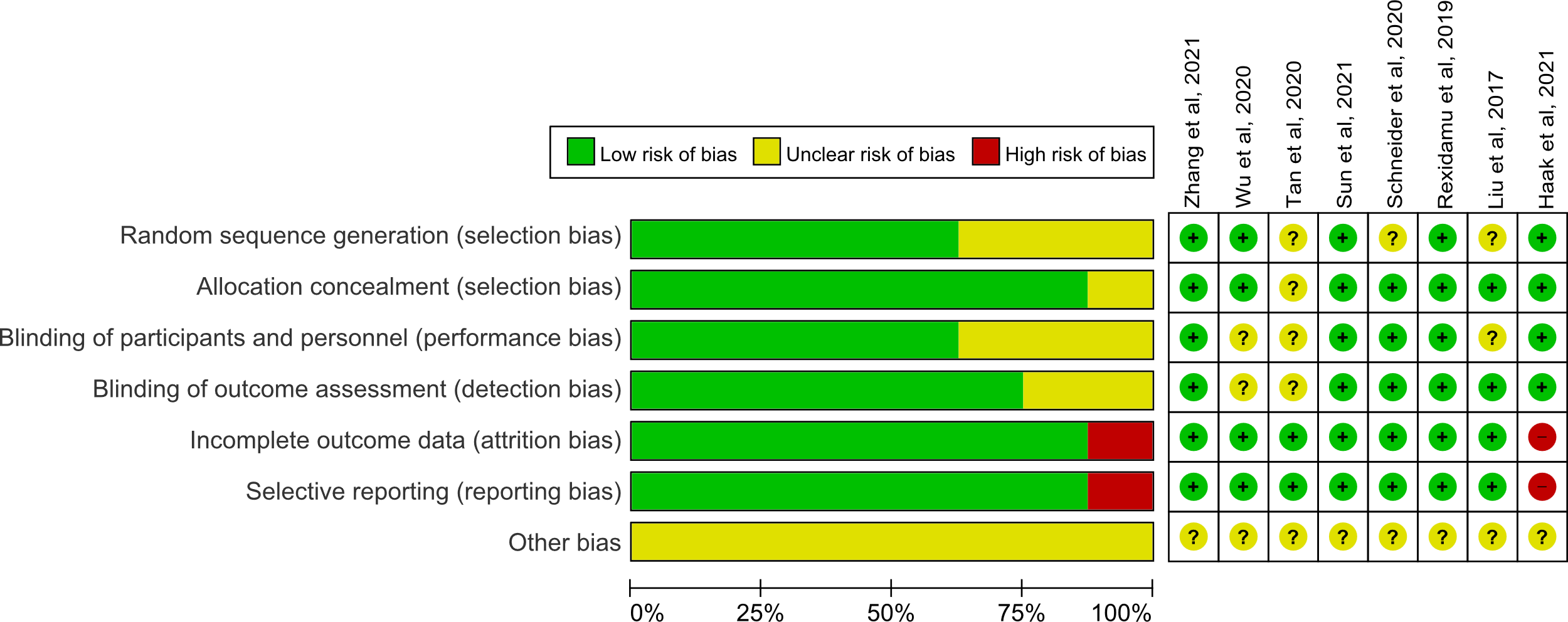


**Fig. S1 Quality assessment of the included studies for meta-analysis.** The evidence levels of identified studies were assessed according to the criteria by the Centre for Evidence-Based Medicine in Oxford, UK [6].


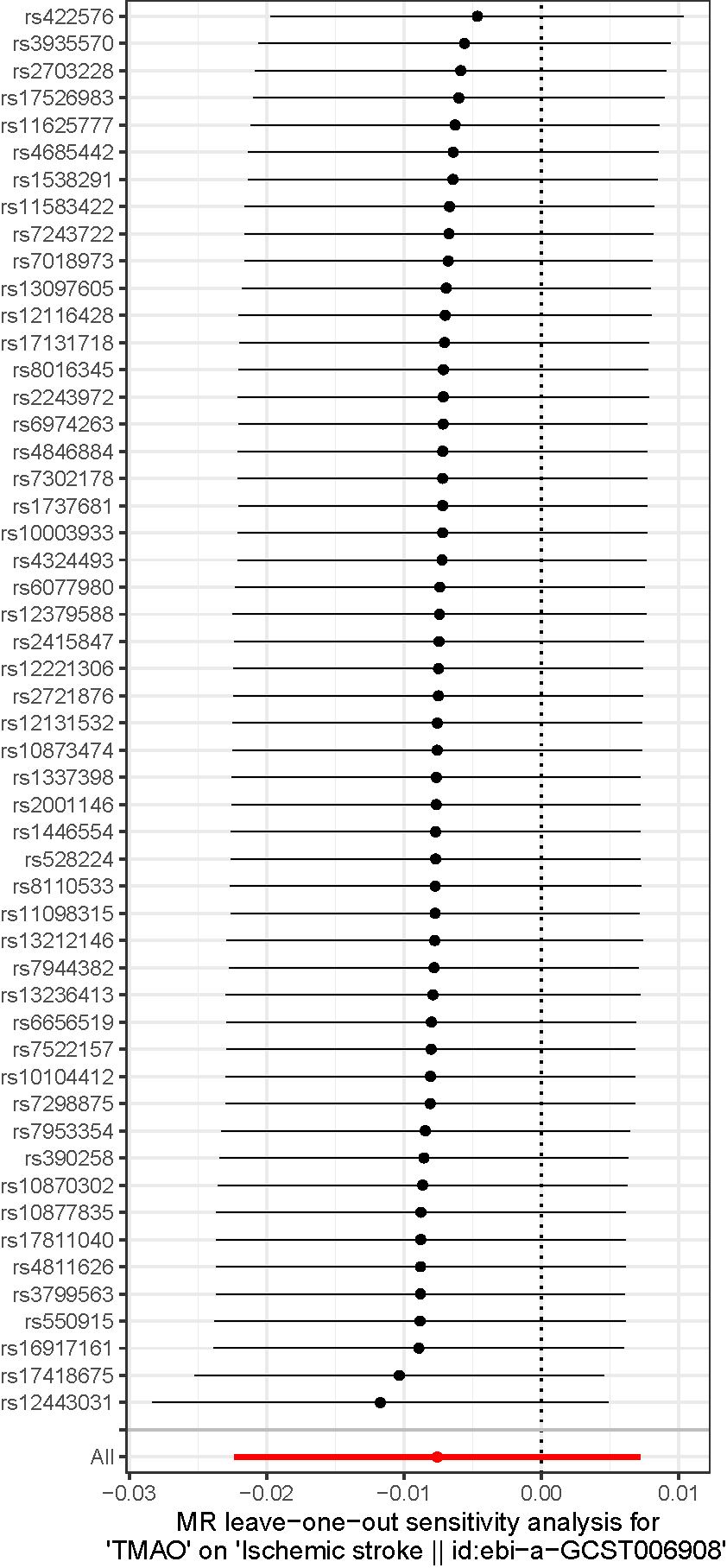


**Fig. S2 Leave-one-out analysis indicated that no single SNP was driving the association of MR analysis.**

**
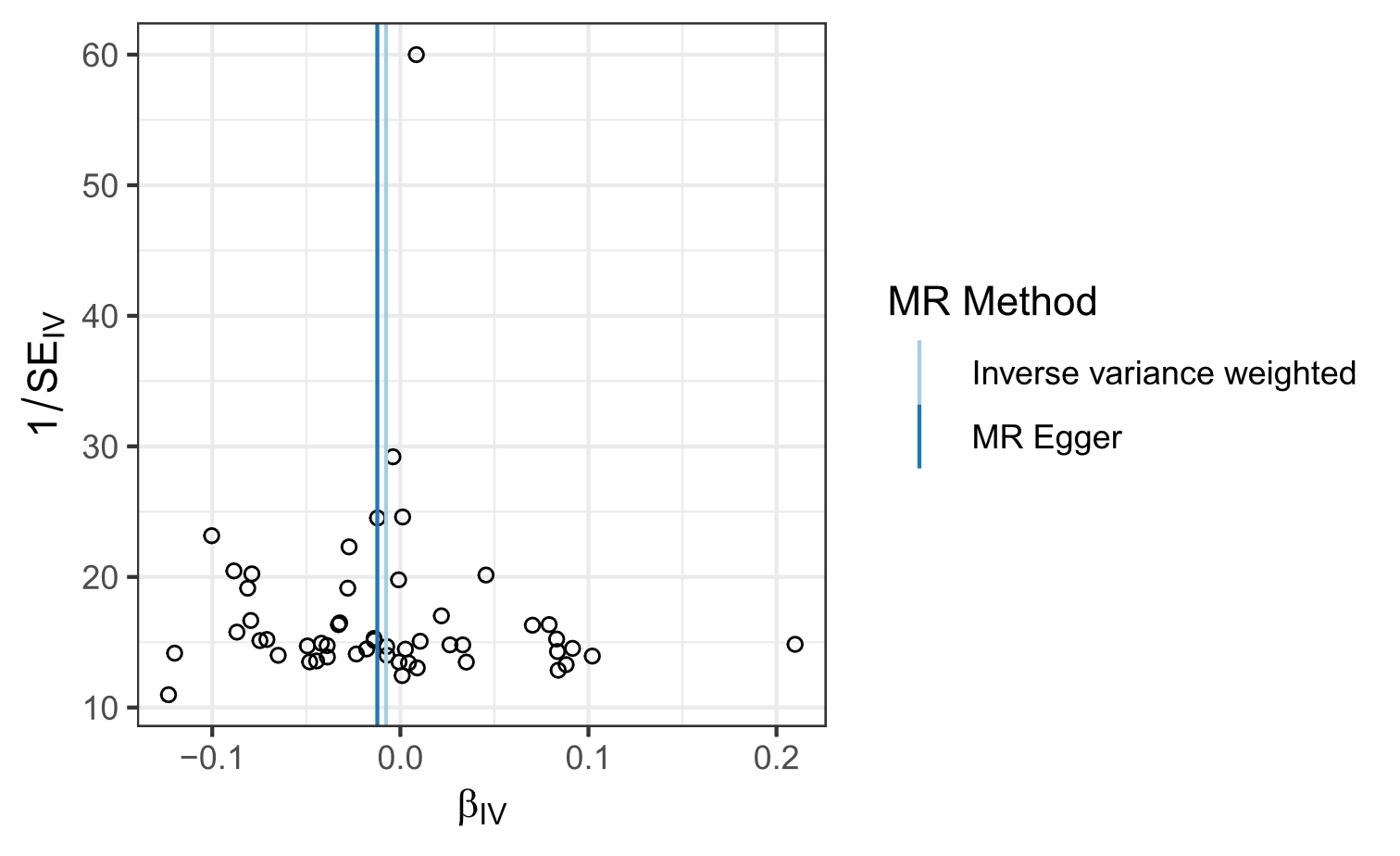
**

**Fig. S3 The funnel plot for visually inspection for horizontal pleiotropy of MR analysis.**

**
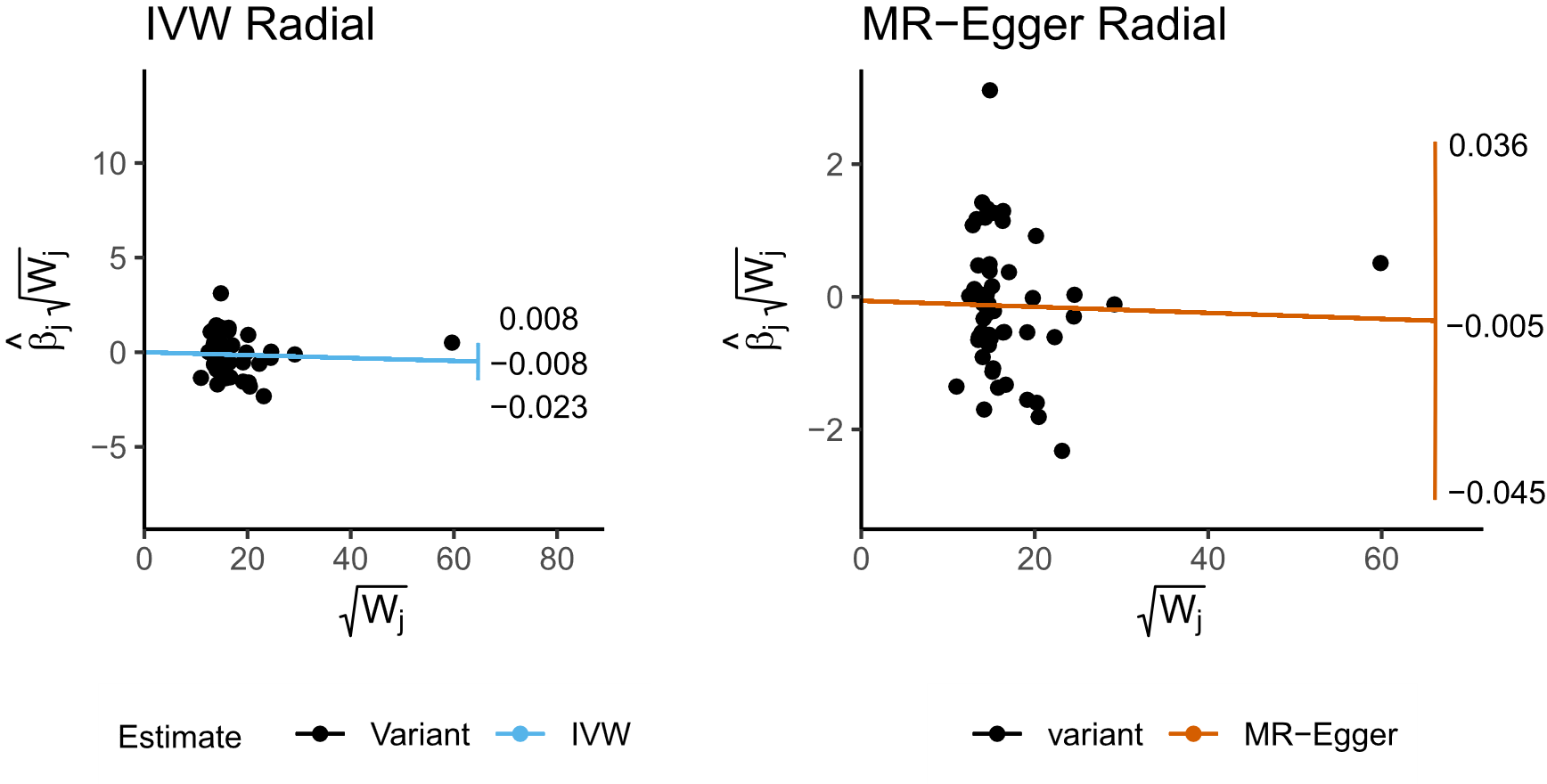
**

**Fig. S4 The Radial plots for classification of outliers in the SNPs.**
